# A Novel High-Throughput Nanopore-Sequencing-Based Strategy for Rapid and Automated S-Protein Typing of SARS-CoV-2 Variants

**DOI:** 10.3390/v13122548

**Published:** 2021-12-19

**Authors:** Gabriel E. Wagner, Massimo G. Totaro, André Volland, Michaela Lipp, Sabine Saiger, Sabine Lichtenegger, Patrick Forstner, Dorothee von Laer, Gustav Oberdorfer, Ivo Steinmetz

**Affiliations:** 1 Diagnostic and Research Institute of Hygiene, Microbiology and Environmental Medicine, Medical University of Graz, 8010 Graz, Austria; michaela.lipp@medunigraz.at (M.L.); sabine.saiger@medunigraz.at (S.S.); sabine.lichtenegger@medunigraz.at (S.L.); patrick.forstner@medunigraz.at (P.F.); 2 Department of Biochemistry, Graz University of Technology, 8010 Graz, Austria; massimo.totaro@tugraz.at (M.G.T.); gustav.oberdorfer@tugraz.at (G.O.); 3 Department of Hygiene, Microbiology and Public Health, Institute of Virology, Medical University of Innsbruck, 6020 Innsbruck, Austria; andre.volland@i-med.ac.at (A.V.); dorothee.von-laer@i-med.ac.at (D.v.L.)

**Keywords:** SARS-CoV-2, next-generation sequencing, vaccine escape, S-protein, nanopore, surveillance, typing

## Abstract

Rapid molecular surveillance of SARS-CoV-2 S-protein variants leading to immune escape and/or increased infectivity is of utmost importance. Among global bottlenecks for variant monitoring in diagnostic settings are sequencing and bioinformatics capacities. In this study, we aimed to establish a rapid and user-friendly protocol for high-throughput S-gene sequencing and subsequent automated identification of variants. We designed two new primer pairs to amplify only the immunodominant part of the S-gene for nanopore sequencing. Furthermore, we developed an automated “S-Protein-Typer” tool that analyzes and reports S-protein mutations on the amino acid level including a variant of concern indicator. Validation of our primer panel using SARS-CoV-2-positive respiratory specimens covering a broad C_t_ range showed successful amplification for 29/30 samples. Restriction to the region of interest freed sequencing capacity by a factor of 12–13, compared with whole-genome sequencing. Using either the MinION or Flongle flow cell, our sequencing strategy reduced the time required to identify SARS-CoV-2 variants accordingly. The S-Protein-Typer tool identified all mutations correctly when challenged with our sequenced samples and 50 deposited sequences covering all VOCs (December 2021). Our proposed S-protein variant screening offers a simple, more rapid, and low-cost entry into NGS-based SARS-CoV-2 analysis, compared with current whole-genome approaches.

## 1. Introduction

Emerging SARS-CoV-2 variants and their rapid spread represent major challenges for global vaccination strategies and health care systems [1,2]. Repeated waves of COVID cases highlight the relevance of tracking SARS-CoV-2 S-protein mutations, because of their importance for vaccine efficacy and therapeutic antibody therapy [3,4]. Thus far, identification of SARS-CoV-2 S-gene variants has relied on next-generation sequencing of the whole genome [5,6,7] or Sanger sequencing of PCR products [8]. RT-PCR has been used for rapid variant identification, screening for certain known mutations only [9,10].

For whole-genome sequencing, nanopore sequencing and the ARTIC pipeline have proven to be powerful tools to monitor genome alterations of circulating SARS-CoV-2 variants [5,6,7]. Here, we propose a nanopore-based strategy to enable fast and easy molecular surveillance at low cost in the diagnostic laboratory, by reducing the sequenced region primarily to the S-protein’s immunodominant S1 subunit (region of interest (ROI)). Furthermore, we provide a user-friendly analysis pipeline for broad application in a diagnostic setting.

## 2. Materials and Methods

DNA quantification mentioned in this publication was carried out using the Qubit dsDNA HS Assay Kit on a Qubit 4 fluorometer (Thermo Scientific, Waltham, MA, USA).

SARS-CoV-2 RNAs were isolated from nasopharyngeal or throat swabs using the MagMAX Viral/Pathogen Nucleic Acid Isolation Kit (Thermo Scientific) on a KingFisher Flex (Thermo Scientific Thermo Scientific, Waltham, MA, USA) according to manufacturer instructions.

The reported C_T_ values were obtained using the Cobas SARS-CoV-2 Kit on a Cobas 6800 system (Roche Diagnostics, Vienna, Austria), according to manufacturer instructions.

Sample amplification and library preparation for nanopore sequencing were carried out as described in the “PCR tiling of SARS-CoV-2 virus with rapid barcoding” protocol, based on a rapid barcoding kit (Oxford Nanopore, Oxford, UK). However, before barcoding, samples were adjusted to the same DNA concentration and, if stated, our S1-ROI primer panel (Table 1) was used for amplicon generation.

To assess the factor of sequencing time reduction, we used the S1-ROI panel and the whole-genome “midnight” panel [11], respectively, to amplify an otherwise identical sample from the same starting material. Subsequently, the amplified DNA was subjected to another PCR using the respective primer panel to generate two equally concentrated pools with sufficient material for the downstream experiment. Therefore, both pools were adjusted to 30 ng/µL, and each pool was used to generate 48 barcoded samples. The resulting 96 samples were sequenced as described below.

All libraries were sequenced on R9 flow cells on a MinION (Oxford Nanopore, Oxford, UK). Sequences were analyzed using the ARTIC pipeline [7,12] in combination with our newly developed S-Protein-Typer tool.

When the DNA sequences were fed to the S-Protein-Typer, it performed a translation and alignment operation, before identifying the amino acid differences, compared with the wild-type strain, and reporting them. The resulting mutations were then evaluated by a random forest classifier, which matched them with any of the known variants of concern (VOC).

Files for processing sequence data resulting from our primer panel, the developed S-Protein-Typer tool, and instructions for installation are available at https://github.com/MassimoGregorioTotaro/s-protein-typer (accessed on 18 December 2021).

## 3. Results

**Figure 1:**
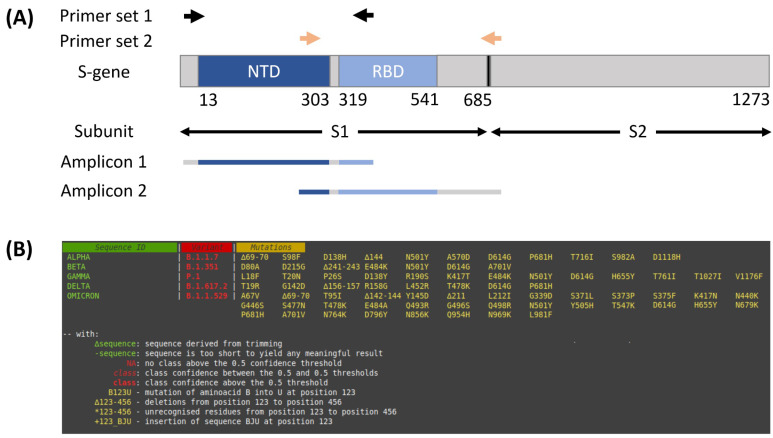
(**A**) Schematic representation of the S-gene and the corresponding subunits. The N-terminal domain (NTD) is shown in dark blue, the Receptor-binding domain (RBD) in light blue. Primer binding sites of the S1-ROI primer panel and amplicons are indicated as follows: black arrows—primers for amplicon 1, red arrows—primers for amplicon 2; (**B**) results as reported by the developed S-Protein-Typer. The columns represent isolate name (green), VOC indicator (red), and amino acid mutations (yellow) compared to the wild-type strain Wuhan-Hu-1.

In order to use nanopore sequencing for molecular surveillance of the S-protein’s immunodominant part (region of interest), we designed a new panel “S1-ROI” consisting of four primers only (Figure 1A and Table 1). The sequenced part of the generated amplicons spans its amino acids 15-683, which are the target region of more than 90% of neutralizing antibody activity [4]. Notably, our panel is suitable for Sanger sequencing due to a 209 base pair (bp) overlap between the amplicons.

We first validated our panel using 30 SARS-CoV-2-positive respiratory samples (C_T_ range between 18.14 and 28.25). All except one sample (C_T_ 27.01) were successfully amplified.

We then demonstrated the gain of sequencing capacity, which enables either an increase in the number of samples and/or a decrease in the required sequencing time. Sequencing time reduction was assessed by sequencing a balanced pool of 48 S1-ROI and 48 “midnight” panel samples on the same MinION flow cell (Table 2). No significant difference (*p* = 0.99, *t*-test) in the mean output per sample was observed. However, an average of 603.4 kbp results in a target coverage of ~265× in the case of the S1-ROI panel, whereas the 602.8 kbp in the case of the whole-genome “midnight” panel results in ~20× target coverage, respectively. This leads to a ~13× higher sequence coverage using the S1-ROI panel.

Sequencing six patient samples (C_T_ 18.14–25.91) using both panels on the MinION flow cell (*p* = 0.62, *t*-test) and the significantly cheaper but also lower output Flongle flow cell (*p* = 0.28, *t*-test) again showed no significant difference in the output and a ~12× higher coverage of our panel (Table 2).

For automated analysis of S-protein amino acid mutations, we developed a versatile tool (Figure 1B) that can handle data regardless of the sequencing technology used and that can be adapted to any viral protein with minor changes in the codebase. As input files, single and concatenated sequences in FASTA format are accepted. These are extensively elaborated before translation to ensure analytical robustness. Once the reading frame is identified, the mutations and predicted VOC are presented to the user. Our tool correctly identified all mutations of the respective variants when challenged with data from our collection and 50 deposited sequences, 10 of each VOC (as of December 2021: Alpha, Beta, Gamma, Delta, and Omicron); see Supplementary Material for corresponding NCBI Sequence Accession numbers.

## 4. Discussion

Here, we report a rapid and cost-saving approach for the detection of SARS-CoV-2 S-protein variants, by nanopore sequencing, based on sequencing only an ROI rather than the whole genome. Given that currently authorized SARS-CoV-2 vaccines rely almost exclusively on the S-protein’s antigenicity and the associated immune protection, the surveillance of its variants is of importance due to high relevance for potential vaccine escape and/or infectivity [13,14].

The observations summarized by Harvey et al. [4], indicating that most epitopes for neutralization belong to the receptor-binding domain (target of 90% of neutralizing antibody activity) and the amino-terminal domain, prompted us to design a specific “S1-ROI” primer set consisting of just two primer pairs. Amplicon length and overhangs also allow for Sanger sequencing, should a MinION be unavailable or time not be a priority.

Our ROI-focused approach frees sequencing capacity per isolate due to the reduction in the sequenced region, hence no sequencing capacity “is lost” to other parts of the genome. Therefore, as the DNA amount per flow cell is fixed, one can (I) increase the number of samples or (II) reduce sequencing time. This is demonstrated by our proof-of-concept experiments with balanced samples, showing that it is possible to obtain the same coverage by a factor of 13 faster using our panel. Moreover, our primer panel cuts initial cost substantially compared to the whole-genome panels (e.g., 4 primers instead of 58 “midnight” panel primers).

Validating our approach with six patient samples on a MinION and Flongle flow cell, respectively, showed very similar results with a factor of reduction of ~12× in both cases. The Flongle is the cheapest nanopore flow cell but comes with the lowest sequencing capacity. Nevertheless, the overall Flongle output (20.2 Mbp) we obtained would allow for 40 samples with 200× coverage, effectively reducing its price/sample to ~1.5 EUR. 

Notably, these advantages come at the cost of sacrificing information about mutations in other parts of the genome, which might contribute to virulence, transmissibility, etc. In such a case, however, the ROI can easily be extended by designing additional primers to complement our panel.

A straightforward and intuitive analysis of sequencing results is key for a broad application. Our automated tool identifies S-protein mutations, compared with the wild-type, and reports the alterations on the amino acid level. Thereby, the evaluator can easily spot combinations of adverse mutations in case of newly emerging variants. In combination with tools such as InterARTIC [15], easy reconstruction and analysis of the S-protein sequence can be obtained with minimal bioinformatics expertise once the pipeline is set up.

In conclusion, our strategy enables low threshold access to fast molecular surveillance of SARS-CoV-2 S-protein variants with regard to cost and bioinformatics knowledge. Such strategies will be essential during the next waves of the pandemic to intensify sequencing efforts for close monitoring of circulating variants, in Europe and especially in lower-income countries. Ultimately, the workflow and tools described can easily be adapted and implemented for other viruses, rendering it a general strategy for surveillance of variants of concern.

## Figures and Tables

**Table 1:** S1-ROI primer set: Primer names and sequences to amplify the region of interest (immunodominant domain of the S-protein). Furthermore, the amplified region of the reference strain (Wuhan-Hu-1) and the factor of reduction, compared with whole-genome amplification, and hence theoretical reduction in sequencing time are denoted.

S1-ROI Primer Panel	
S1-ROI-1_Forward	TGCCACTAGTCTCTAGTCAG
S1-ROI-1_Reversed	CACAGTTGCTGATTCTCTTCC
S1-ROI-2_Forward	CAGATGCTGTAGACTGTGC
S1-ROI-2_Reversed	TGACTAGCTACACTACGTGC
Amplified region	21,585–23,631
Factor of reduction compared with the whole genome	~13.1

**Table 2:** Sequencing summary of nanopore runs comparing our S1-ROI primer panel and the whole-genome “midnight” panel suggested by Oxford Nanopore: (A) a 20 min MinION run of a balanced pool of 48 S1-ROI and 48 Midnight samples each to assess the factor of time reduction; (B) six patient samples were sequenced with both panels on a MinION for 15 min to ensure applicability of the approach in case of original respiratory samples. Sequencing time was reduced compared to (A) due to the lower number of samples; (C) the library used in (B) was also sequenced on a Flongle to reconfirm the results in this setting and to assess overall Flongle output. As Flongle outputs are generally much lower, the flow cell was run till the end (18 h). Sequencing coverage was calculated by dividing the mean output per sample by the respective target (S1-ROI: 2.275 kbp; Midnight: 29.903 kbp). The factor of S1-ROI sequencing time reduction (sequencing coverage S1-ROI/sequencing coverage midnight), exemplifies the time reduction to obtain the same coverage.

	(A) MinION Balanced Samples		(B) MinION Patient Samples		(C) Flongle Patient Samples	
Primer panel	S1-ROI	Midnight	S1-ROI	Midnight	S1-ROI	Midnight
Number of samples	48	48	6	6	6	6
Mean output per sample [kbp]	603.44	602.82	4225.80	4656.64	1576.81	1792.32
SD [kbp]	257.29	257.18	1162.81	1541.23	420.77	355.29
Sequencing coverage	265	20	1857	156	693	60
Factor of S1-ROI sequencing time reduction	13		12		12	

## Supplementary Materials

The following are available online at https://www.mdpi.com/article/10.3390/v13122548/s1, Table S1: NCBI sequence accession numbers used for validation of the S-Protein-Typer.
